# Pathogenesis, Prophylaxis, and Treatment of *Candida auris*

**DOI:** 10.3390/biomedicines12030561

**Published:** 2024-03-01

**Authors:** Madalina Preda, Razvan Daniel Chivu, Lia Mara Ditu, Oana Popescu, Loredana Sabina Cornelia Manolescu

**Affiliations:** 1 Department of Microbiology, Parasitology and Virology, Faculty of Midwives and Nursing, “Carol Davila” University of Medicine and Pharmacy, 020021 Bucharest, Romania; madalina.preda@umfcd.ro (M.P.); loredana.manolescu@umfcd.ro (L.S.C.M.); 2 Clinical Laboratory of Medical Microbiology, Marius Nasta Institute of Pneumology, 050159 Bucharest, Romania; 3 Department of Public Health and Health Management, Faculty of Midwifery and Nursing, “Carol Davila” University of Medicine and Pharmacy, 050474 Bucharest, Romania; 4 National Institute for Research & Development in Chemistry and Petrochemistry—ICECHIM Bucharest, 202 Spl. Independentei, 060021 Bucharest, Romania; lia_mara_d@yahoo.com; 5 Faculty of Biology, University of Bucharest, 90 Panduri Street, 050663 Bucharest, Romania; 6 National Reference Laboratory of Tuberculosis, Marius Nasta Institute of Pneumology, 050159 Bucharest, Romania; oana.popescu@marius-nasta.ro

**Keywords:** *Candida auris*, fungal pathogen, emerging threat, diagnosis, antifungal resistance

## Abstract

*Candida auris* poses a serious threat to infection control and patient care since it can produce invasive infections that have a high fatality rate, has been linked to outbreaks in hospital environments, and is typically resistant to several antifungal medications. Since its first description in 2009, six clades have been described. The emerging fungal pathogen possesses adhesins that allow it to adhere to host tissues and medical devices, can form biofilms, produces various hydrolytic enzymes, employs several strategies to evade host immune responses, and exhibits high genetic diversity, which may contribute to its ability to adapt to different environmental conditions and evade host defenses. *C. auris* is very resistant to various disinfectants and may be difficult to detect.

## 1. Introduction

*Candida auris* is an increasingly dangerous fungus that poses a threat to world health [1]. Deep-seated infections and devious hospital outbreaks may be caused by *C. auris* [2]. It is mostly collected from hospital surroundings, and because of its special characteristics, it can colonize and remain for extended periods in both patients and hospital settings, which can lead to a vicious cycle of infection, acquisition, and spread, especially in intensive care units [2]. It meets all the requirements to be categorized as an urgent public health threat, including the capacity to spread quickly through horizontal transmission, the potential to infect susceptible people with serious and potentially fatal infections, and the unfavorable profile of antifungal agent resistance, in addition to the dearth of standardized preventive and control measures and effective treatment options [3,4].

Since *C. auris* has distinct qualities that increase its capacity for invasion, support resistance to antifungals, and provide it with an increasing advantage in host and ecological habitats, its pathogenicity and virulence are extremely concerning [5]. It has been established that filamentation, biofilm development, osmotolerance, thermotolerance, and the synthesis of hydrolytic enzymes are essential elements of the pathogenesis of *C. auris* [5].

Effective therapy techniques against this disease are urgently needed, as there is currently a concerning dearth of identification strategies accessible worldwide despite its widespread proliferation [6]. Due to challenges in accurately identifying *C. auris* strains, high misidentification rates might also be attributed to a lack of identification strategy [6]. The mode and speed of *C. auris* transmission among healthcare workers and immunosuppressed hospitalized patients can be explained by its high rate of antifungal drug resistance, its ability to colonize the skin and other bodily sites, and its ability to live on abiotic surfaces and equipment for weeks [6]. This is just one of the four unsettling aspects of this fungus [6].

We performed a narrative review to offer an updated image of the current knowledge on *C. auris*, concentrating on aspects such as clinical and microbiological traits, virulence and antifungal resistance mechanisms, and the effectiveness of current control, prophylactic, and treatment approaches.

## 2. Emerging Threat

*C. auris* is linked to nosocomial infections and poses a major risk to public health [7]. It exhibits multi-drug-resistance patterns to typical antifungal medication used for other invasive Candida infections, along with other virulence factors [7]. The first report of *C. auris* was in Japan. The strain was isolated from the external ear canal of a Japanese patient, aged 70, who received treatment at Tokyo Metropolitan Geriatric Hospital in Tokyo [8]. *C. auris* is a member of the ascomycetous (hemiascomycetes) Clavispora clade in the Metschnikowiaceae family of the order Saccharomycetales [9]. These yeasts reproduce by budding.

This strain shares a tight phylogenetic relationship with *Candida ruelliae* and *Candida haemulonii* in the Metschnikowiaceae clade, according to analyses of the 26S rDNA D1/D2 domain, nuclear ribosomal DNA ITS region sequences, and chemotaxonomic studies [8]. Although the first literature report was published in 2009, in a study on stored unidentified yeast samples from fungemia patients, one patient with *C. auris* was diagnosed in a sample stored from 1996 [10].

Initially, four primary clades were identified by genetic studies and were designated after their different geographical locations: clades I, II, III, and IV in South Asia, East Asia, Africa, and South America [11]. In 2018, a fifth clade was suggested through the identification of the first case in Iran [12], which was later confirmed through whole genome sequencing [11]. In 2023, a sixth clade was described in Singapore, which is separated from all other existing clades (I–V) by >36,000 single nucleotide polymorphisms [13].

Numerous specimen types, such as typically sterile bodily fluids, pulmonary sections, urine, bile, tissues, wounds, and mucocutaneous swabs, have yielded clinical isolates of *C. auris* [14].

One of the most notable changes linked to the emergence of the disease related to *C. auris* is that pathogenic isolates seem to have simultaneously emerged independently on three continents [15]. The idea that *C. auris* was an environmental fungus until recently is supported by the fact that it cannot grow anaerobically and is usually found in colder skin areas rather than in the gut [16]. There could have been several non-exclusive causes at play when *C. auris* first appeared 10 years ago [16]. For instance, *C. auris*’s virulence, temperature tolerance, osmotic stress tolerance, and multidrug resistance may be explained by its constitutive overexpression of HSP 90 [15,16]. Thus, *C. auris* might have historically evolved as a plant saprophyte in specific environments, such as marshes [15,16]. Initially, its appearance could have been connected to the consequences of global warming on wetlands, particularly climatic cycles [15,16].

A significant increase in new instances of colonization and infection has been detected as a result of the coronavirus disease 2019 (COVID-19) pandemic, which has altered the landscape of *C. auris* illness [2,17]. This increase is mostly attributable to the overburden of global healthcare systems and the resulting weakening of infection prevention and control procedures [2,18].

## 3. Pathogenesis

The rise of *C. auris* has been attributed in part to the extensive use of antifungal medications [15]. Drug resistance in this fungal species can undoubtedly be attributed to selection by ambient azole use, but this does not explain why the organism rapidly spread to three continents and became a human disease [15]. Long before *C. auris* showed up, azole-resistant Candida species started to arise [15]. It is highly unlikely that a microbe will become pathogenic simply by developing drug resistance, as virulence and decreased drug susceptibility are two very different traits, as shown by the frequent fitness costs linked to mutations in Candida that confer resistance to antifungals [15].

In addition to showing notable intraspecies variability, *C. auris* differs significantly from other species, indicating the need for including as many strains as feasible, as well as strains from the many recognized clades [19]. Thus, the level of pathogenicity of *C. auris* to other species remains unclear to this day. Furthermore, there are still improvable numbers of in vivo investigations and strains utilized in the literature [19]. Moreover, certain research works exhibit methodological constraints that limit their external validity [19].

### 3.1. Biofilm

One of the primary pathogenic characteristics is the production of biofilms [20]. The majority of clinical isolates and colonizing *C. auris* show biofilm development that is comparable to or higher than that of *C. albicans* [20]. Transcriptome research revealed that during the formation and maintenance of biofilms, *C. auris* upregulates adhesin proteins CSA1, IFF4, PGA26, and PGA52 [21]. Major facilitator superfamily proteins MDR1 and RDC3 are increased, while ABC transporter proteins such as CDR1, SNQ2, and YHD3 activate as the biofilm ages [21]. Additionally, within the biofilm, adherence factors facilitate cell-to-cell attachment. Transcriptomic investigations of *C. auris* have led to the hypothesis that the ALS proteins ALS1 and ALS5 play a role in the adhesion of biofilm formation in the bacterium [21].

The majority of colonizing isolates form a biofilm of aggregative pattern [22]. *C. auris* demonstrated phenotypic diversity, with non-aggregative isolates being more common among candidemia patients [22]. This could help to partially explain the organism’s extraordinary capacity to colonize hospital furnishings, surroundings, and human body sites over time, suggesting a possible role for biofilm formation [22,23]. Moreover, the ability to form biofilm increases the ability to resist decolonization methods.

An essential first stage in biofilm production and skin colonization is surface adherence [24]. The functions of certain adhesins for *C. albicans* and other Candida species have been extensively documented [24], while for *C. auris* are still described, and some seem to be specific, for example, Surface Colonization Factor (Scf1) and a conserved adhesin, Iff4109 [25]. While hydrophobic interactions are the mode of action for regular fungal adhesins, Scf1 depends on exposed cationic residues for surface association [25].

Numerous case studies have demonstrated that biofilms play a role in the nosocomial spread of infections [26]. Skin colonization or surface contamination during healthcare provider–patient contact has found healthcare workers to be major *C. auris* vectors [26]. *C. auris* has an increased resistance to disinfectants, especially when forming biofilms. In one study, 13 frequently used hospital disinfectants were tested against *C. auris* biofilms [27]. In total, 58% of the products were unable to stop the spread of *C. auris*, 50% of the products were unable to stop cell viability, and 75% of the disinfectants were unable to stop biofilm regrowth [27].

Since most disinfectants may be inefficient, photodynamic therapy was tested in various studies. The germicidal properties of a decontamination device generating UV-C radiation at a wavelength of 254 nm were evaluated concerning its capacity to penetrate *C. auris* colonization [28]. The robustness of this pathogenic organism is further demonstrated by the fact that *C. auris* growth was not significantly reduced by a 10 min exposure interval, in contrast to *C. glabrata* and *C. albicans* [28]. It took 20 to 30 min of exposure to drastically lower the growth of *C. auris* [28].

### 3.2. Filamentation

Microbial infections frequently employ phenotypic plasticity as a tactic to adjust to a variety of host settings [29]. It was also discovered that genes connected to the cell wall or cell surface expressed differently, suggesting that variations in cell surface antigens are linked to the capacity for filamentation [29]. In pathogenic fungi, morphological plasticity is essential for both virulence and response to environmental changes [30]. Filamentous growth is regarded as a pathogenicity feature in pathogenic fungi and facilitates the exploration of novel settings [31]. Nevertheless, *C. auris* does not develop filamentous growth in response to most of the stimuli that cause filamentation in the most well-studied and distantly related pathogen, *C. albicans* [31]. The growth of pseudohyphae, as opposed to real hyphae, has been linked to a delay in the advancement of the cell cycle and an extension of the apical growth period [31]. It has been shown that medications that cause genotoxic stress, like methyl methanesulfonate or hydroxyurea, cause S phase arrest by way of a cell cycle checkpoint [31]. According to a study, many clinical isolates of *C. auris*, albeit not all of them, can develop into pseudohyphal colonies when exposed to genotoxins like HU, MMS, or the therapeutically useful fungistatic 5-fluorocytosine [31].

There are various cellular morphologies in *C. auris* [30]. The filamentous phenotypes of *C. albicans* and *C. auris* share phenotypic similarities [30]. The ability of *C. auris* to undergo morphological transformations, when paired with its antifungal resistance qualities, may play a role in the rise and fast prevalence of the species worldwide [30].

### 3.3. Signaling Pathways

Despite its significance for world health, *C. auris*’s virulence mechanism and associated signaling pathways are still mostly unknown [32]. The evolutionarily conserved cyclic AMP (cAMP) pathway is one of the recognized signaling pathways that is often necessary for the pathogenicity of plant and human fungal diseases [32]. The cAMP/PKA pathway controls the pathogenicity, biofilm formation, vegetative and hyphal development, and stress response in *C. albicans*. Furthermore, the expression of the genes CDR, MDR, ERG, and TAC1—which are linked to Candida species’ resistance to antifungal medications—is regulated by cAMP [32].

In *C. auris*, KA regulates both Cyr1-dependent and -independent processes, including temperature-dependent growth, pseudohyphae production, stress responses, ploidy switching, and resistance to antifungal medications and disinfectants [32]. Above all, the virulence of *C. auris* was considerably reduced by hyperactivation of the cAMP/PKA pathway but not by inhibition of the route [32].

### 3.4. Enzymes

Of the encoded enzymes in *C. auris*, hydrolases make up 42% of the enzymes that are released. In the genome of *C. auris*, orthologous genes for four secreted aspartyl proteinases (SAPs) have also been found [26]. SAPs are one of the primary contributory factors of Candida’s pathogenicity [33]. SAP production varies among *C. auris* strains and is influenced by environmental conditions [24].

Reduced SAP formation at lower temperatures may be advantageous to reduce immune responses during long-term persistence on the skin because of the skin’s lower surface temperature [34]. Furthermore, SAP production by *C. auris* has been reported at temperatures as high as 42 °C [34]. This indicates a function for SAPs in warm environmental circumstances and is consistent with its observed thermotolerance [34].

Phospholipases can also be secreted by *C. auris*, but this capacity varies according to the strain and is only shown in up to 37.5% of isolates [26]. By contrast, 64% of strains of *C. auris* are capable of producing proteinases. Additionally, except for the CBS 12770 *C. auris* strain, *C. auris* phospholipases are often weaker than *C. albicans* phospholipases [26].

Active hemolysin enzymes found in the majority of *C. auris* strains aid in the competitive sequestration of iron for accelerated growth and dissemination [35]. In addition, *C. auris* reacts to temperature stress more violently than *C. albicans*; at 42 °C, *C. auris* produced more protective aspartyl proteinase than *C. albicans* [35].

Extracellular vesicles (EVs), structures of lipid bilayer-enclosed cargo that influence morphologic changes, host interactions, and drug resistance, are secreted by a variety of fungus species, including *C. albicans* [24,36]. While *C. auris* creates vesicles during planktonic and biofilm modes of growth, some of the cargo and characteristics are different from those of *C. albicans* [24,36].

Sterols, RNA, proteins, and lipids are all present in vesicles; however, the precise contents examined by proteomics and lipidomics differed considerably in *C. auris* compared to *C. albicans*, indicating that these components’ functions might also differ [36]. In a study, *C. albicans* EVs did not increase fungal adherence to epithelial cells, while *C. auris* EVs could [36]. It was discovered that EVs from the two *C. auris* isolates stimulated murine bone marrow-derived dendritic cells by upregulating the expression of costimulatory molecules and MHCII in a manner akin to that of *C. albicans* [36].

Transmission Electron Microscopy revealed that the electron-dense pigmentation-related regions are absent from *C. auris* EVs [36]. When adjusted for the quantity of generating cells, the total protein and ergosterol concentrations in EV suspensions were greater in *C. albicans* than in either *C. auris* strain [36]. Regardless of the number of EV-producing cells, the primary components of EVs may be measured using the ratio between the concentrations of protein and sterol, and *C. auris* MMC2 had a greater ratio than *C. albicans* or *C. auris* MMC1 [36].

## 4. Disease and Immune Response

Recently, there has been a rise in the occurrence of opportunistic fungal infections, which are often not disease-causing in healthy individuals [37]. They may complicate other diseases and may pose a threat to people with underlying medical conditions, immunocompromised individuals, those who take antibiotics often, and those suffering from pulmonary tuberculosis [37]. Lesions can be localized to the lung apex or spread throughout the entire lung [38]. There may be pleura, lung parenchyma, or bronchial airway damage associated with tuberculosis [38]. When lesions impact the pulmonary parenchyma, connective tissue replaces it, changing the architecture of the lungs [38]. The result is fibrosis, or total lung deterioration, and the formation of scars or resting cavities, which are usually bounded by long-standing fibrous parenchyma strips [38].

Other diseases with a risk of fungal infectious complications include chronic obstructive pulmonary diseases, which can lead especially to exacerbations [6,7,39]. A worsening of three or more of the primary symptoms, cough sputum consistency and/or volume, for longer than 48 h is considered an exacerbation of bronchiectasis [40].

Patients with serious underlying medical disorders who require complex medical care are primarily affected by *C. auris* [1,41]. Individuals who have invasive medical devices such as urine catheters, feeding tubes, breathing tubes, or venous catheters are more likely to contract *C. auris* and become infected [1,42]. Healthy individuals without these risk factors, such as family members and healthcare professionals, have a low chance of contracting *C. auris* (Table 1) [1].

Other risk groups may include people who use drugs or alcohol excessively, those in prison or under enforced segregation, certain vulnerable immigrant populations who are denied access to health and social care services, and other marginalized, impoverished, and remote groups and difficult-to-reach populations [41,42].

*C. auris* can lead to infections in the bloodstream, ears, and open wounds, among other areas of the body [43]. The location and degree of the *C. auris* infection determine the symptoms [43]. At the moment, a typical set of symptoms unique to *C. auris* infections does not exist. The symptoms could resemble those of another *Candida* spp. or a bacterial infection (Table 2) [43].

A carefully balanced interaction between innate and adaptive immune responses is necessary for host defense against Candida species [44]. First, the skin and mucosa form a physical barrier [44]. The ability of innate immune cells like neutrophils, macrophages, and monocytes to recognize pathogen-associated molecular patterns, or components of the fungal cell wall that have evolved through evolution, is a major prerequisite for the second barrier, which is represented by the innate immune system [44]. To shape adaptive immunity, a long-term defense against fungal infection, pro-inflammatory cytokine production in conjunction with myeloid cells’ antigen-presenting ability is essential [44].

In different studies, a reduced ability to destroy and phagocytose *C. albicans* was observed. It was shown that *C. auris*’s poor neutrophil phagocytosis was preserved across a range of strains and clades [45]. In contrast to the reaction to *C. albicans*, *C. auris* did not cause the development of neutrophil extracellular traps or produce significant levels of reactive oxygen species [45].

It seems that *C. auris* uses a variety of strategies to evade the immune system [46]. According to a recent study looking at the interactions between *C. auris* and macrophages, *C. auris* might elude macrophages generated from mouse bone marrow following phagocytosis [46]. It was demonstrated that *C. auris* could effectively reduce macrophage glucose concentrations and cause their death after intracellular replication without triggering inflammatory reactions in the cells [46]. Infectious stimuli can cause the bronchiectatic airway epithelium to overreact with pro-inflammatory cytokines [47]. Interleukin (IL)-6 and persistent airway inflammation are two of the mediators linked to the control of inflammation in bronchiectatic airways [47]. Compared to bone marrow-derived macrophages infected with *C. albicans*, those infected with *C. auris* produced less IL-1β, a cytokine that is associated with lower inflammasome activation [46,47].

Mannosylation of the cell wall of *C. auris* affects neutrophil interactions, which encourages neutrophil evasion in this species [45]. Mutations in the mannosylation pathways confer enhanced phagocytosis and cytotoxicity upon human neutrophils [45]. Additionally, it was discovered that zebrafish phagocytosis and neutrophil recruitment are impacted by *C. auris* mannosylation [45].

## 5. Prevention

Microorganisms in both patient and environmental collections are frequently complex communities [48]. There is mounting evidence that *C. auris* can colonize hospital surroundings over time and is more likely to spread in healthcare settings [48,49]. Since *C. auris* is frequently resistant to numerous antifungal medication classes, infection prevention in healthcare settings led by rapid identification is crucial [50]. Inadequate screening protocols may result in misdiagnosis or delayed diagnosis, increased rates of transmission and mortality, and severe cost implications [50].

The remarkable characteristic associated with the establishment of *C. auris* as a nosocomial pathogen is its capacity to last on abiotic hospital surfaces, even in the face of strict cleaning practices [9]. The esterase activity assay, which checks each cell for viability, showed that *C. auris* cells were viable on surfaces for an additional two weeks after what was found by culture in a study on the survival of *C. auris* on objects where culturable cells were retrieved up to that point [48]. After contamination, viable *C. auris* can be found via esterase activity or recovered from plastic surfaces up to two and four weeks later, respectively [48]. These results raise the intriguing question of whether *C. auris* can develop into viable but non-culturable cells that last in medical settings [9]. The stress tolerance and persistence of *C. auris* can be inferred from current metabolomic, transcriptomic, and molecular research, even though no studies have directly addressed this subject [33].

Culture-positive skin, oropharynx, vascular line exit site, respiratory tract, and urinary tract without clinical evidence of Candida infection may be considered colonization with *C. auris* [51].

In healthcare workers in contact with *C. auris*-positive patients, screening procedures may be required in regions like the hands, nose, axilla, groin, and throat swabs. Decolonization procedures for contact may include chlorhexidine washes, ointments, and oral nystatin medication [51].

For colonized patients, decontamination procedures may include oral nystatin if oropharyngeal colonization was present, mouthwash containing 0.2% chlorhexidine, or 2% chlorhexidine gluconate washes using single-use wipes or an aqueous formulation of 4% chlorhexidine [51].

Since in vitro studies demonstrate that chlorhexidine gluconate (CHG) inhibits *C. auris* isolates at <0.02%, bathing patients in a 2% solution of the drug is a standard method for cleansing their skin in many clinical settings, including intensive care units [52]. But, even with its in vitro activity, *C. auris* can linger on patients’ skin in medical settings where CHG washing is a regular practice [24]. Regular bathing might not be sufficient to deliver CHG at the necessary concentration to every colonized site [24]. Studies conducted ex vivo indicate that the efficacy of CHG may be enhanced by the addition of 70% isopropyl alcohol and widely used topical essential oils, such as tea tree (*Melaleuca alternifolia*) and lemongrass (*Cymbopogon flexuosus*) oils [53].

Besides treating infections and dealing with the colonization of patients or healthcare workers, surfaces are very important as well. Tests were conducted on the effects of NaOCl and peracetic acid on surfaces made of cellulose, polymers, and stainless steel [54]. On all surfaces, both disinfectants demonstrated notable efficiency in eliminating *C. auris* cells [54]. Nevertheless, after applying NaOCl to non-porous surfaces (polyester coverslips and stainless steel), some live cells were still present [54]. In actuality, longer exposure times combined with larger doses of this disinfectant were necessary to reduce regrowth; yet, even then, the pathogen could not be eliminated [54]. For instance, a five-minute exposure to stainless steel at a concentration of 10,000 parts per million of NaOCl was sufficient to detect a discernible decline in colonies [54]. However, after re-inoculation onto a rich medium, significant regrowth was seen [54].

## 6. Diagnosis

It is important to identify Candida isolates from typically sterile body areas down to the species level so that early medication can be tailored to the patient’s expected sensitivity to the disease [14]. When conventional testing techniques are used in the microbiology lab, *C. auris* is frequently misidentified [26,50]. The diagnosis must be comprehensive and approach multiple directions, for example, other complementary tests, such as C reactive protein, which may be particularly important for tracking the course of the illness or the efficacy of treatment [50]. Due to their increased accessibility and lower cost as blood tests, the usefulness of the neutrophil-to-lymphocyte ratio and platelets-to-lymphocyte ratio as indicators of systemic inflammation has been examined in many studies conducted in recent years [40]. The significance of the platelets-to-lymphocyte ratio remains even if the neutrophil-to-lymphocyte ratio is a valid inflammatory marker in solid tumors, bronchiectasis, sleep apnea, and several other diseases [40].

According to the Centers for Disease Control and Prevention, in specific situations, species-level identification of Candida isolated from non-sterile places should be taken into consideration, such as when clinically indicated in the care of a patient; when an institution or unit has had a case of *C. auris* infection or colonization, further patients may need to be identified to confirm the colonization or when a patient has spent the night in a medical facility outside of the United States in the past year, as some *C. auris* patients have been found to have been colonized for longer than a year [55].

It is possible that the prevalence and geographic scope of *C. auris* illness, which primarily affects low- and middle-income nations, are underestimated [2]. The lack of a worldwide identification plan and the low precision of the current generation of traditional diagnostic instruments are the two main causes of data scarcity [2].

Using selective enrichment broth media that are tailored to the growth parameters of *C. auris* results in enhanced sensitivity and specificity as well as a quicker recovery period for clinical specimens [48]. The odds of isolation may be increased by using a 10% salt Sabouraud Dulcitol broth medium with gentamicin and chloramphenicol, shaking the inoculum, and then incubating it at 37–40 °C [48]. This makes use of *C. auris*’s special capacity to grow in saline environments (10% *w*/*v*) and at high temperatures [48]. While *C. auris* may grow in these settings using dulcitol or mannitol as a carbon source, *C. haemulonii* and *C. duobushaemulonii* cannot grow in these conditions and need glucose as a carbon source (Table 3) [48,56,57].

On Sabouraud dextrose agar, *C. auris* strains have been described as white to cream-colored colonies, whereas on CHROMagar, they are pink to beige [14]. New chromogenic medium formulations have been created especially to detect *C. auris* [26].

The strains grow well at 37 °C and 42 °C and assimilate N-acetylglucosamine, succinate, and gluconate, in contrast with *C. haemulonii* and *C. duobushaemulonii*, which do not grow at 42 °C, nor do they assimilate the previously mentioned sugars [14]. Contrary to the majority of fungi, which cannot endure temperatures below that of human metabolism, *C. auris* demonstrates thermotolerance, enabling it to thrive at temperatures over 37 °C and continue to be viable at temperatures as high as 42 °C [5]. It has a high tolerance for high-salinity conditions (>10% NaCl, *w*/*v*) and osmotic stressors [15]

The budding yeast *C. auris* may be observed in single, paired, or group cells [7]. The cells are 2.5–5.0 μm in size, ovoid, and ellipsoidal to elongate [7]. Rarely, it can undergo filamentation, a crucial stage in the fungal invasion of host tissues, in the same way as other Candida species, producing both pseudohyphae and real hyphae [2]. *C. auris* can create basic pseudohyphae in biofilm development and under high-salinity conditions [26]. Candidalysin (ECE1) and hyphal cell wall protein (HWP1), which are necessary for complete hyphal development, are absent in *C. auris* [33]. *C. auris* can grow filaments at lower temperatures (20 °C and 25 °C), in contrast to *C. albicans*, where higher temperatures cause hyphal development [29].

Most of the time, laboratory diagnosis is also based on the culture aspect of different chromogenic media (Table 4).

When employing conventional phenotypic techniques for yeast identification, such as the VITEK 2 YST, API 20C, BD Phoenix yeast identification system, and MicroScan, *C. auris* can be mistaken for a variety of distinct organisms (Table 5) [1].

When it comes to identifying *C. auris*, matrix-assisted laser desorption ionization-time of flight mass spectrometry (MALDI-TOF MS) is more accurate than other approaches and enables the subsequent epidemiological characterization of strains [26].

To identify *C. auris* nucleic acids, several commercial systems have been developed, showing acceptable sensitivity (89–100%) and specificity (85–100%) [58]. Some molecular assays show suitable diagnostic accuracy when compared to the reference method of culture combined with MALDI-TOF identification, demonstrating its extensive utility for quick surveillance and diagnosis, for example, DiaSorin Molecular Simplexa^®^ Detection Kit [59]. The technology significantly improved its clinical value by providing data two hours after swab collection [59].

## 7. Treatment and Antifungal Resistance

### 7.1. Treatment Options

The potential for these organisms to harbor or develop multidrug resistance is one of the reasons the appearance of *C. auris* has been so concerning [14]. At this point, no susceptibility breakpoints specific to *C. auris* have been identified [1]. As a result, the expertise of professionals and breakpoints developed for related Candida species are used to characterize them (Table 6) [1]. It is currently unknown if microbiologic breakpoints and clinical outcomes are correlated [1].

At this moment, it is unknown what the best course of action is for treating *C. auris*. Initial therapy is advised to include consultation for infectious diseases and treatment with medication from the echinocandin class, as most *C. auris* isolates found in the United States have been susceptible to these drugs (Table 7) [14].

If the patient develops chronic fungemia for more than five days or is clinically unresponsive to echinocandin treatment, switching to liposomal amphotericin B (5 mg/kg daily) may be necessary [60].

Infection control measures may include that all positive patients be isolated [51]. The presence of this yeast should be checked in all direct contact with patients’ noses, axillas, groins, throats, rectums, or feces, as well as clinical samples including urine, wounds, drains, and respiratory collections [51]. The minimum contact period for the acquisition of *C. auris* with a positive case or a contaminated environment is approximately ≥4 h [51]. Some studies recommend that only after three consecutive negative *C. auris* screens should direct contact patients be de-isolated, and then they should be checked every week until their discharge [51].

### 7.2. Resistance Mechanisms

**Figure 1:**
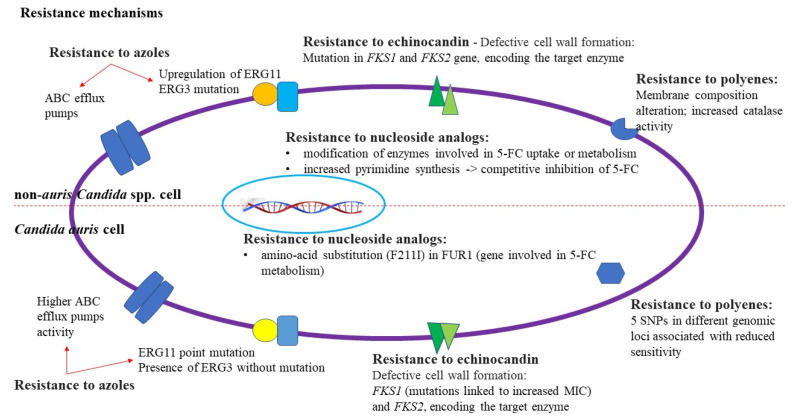
*Candida* spp. and *C. auris* resistance mechanisms [9,53].

Drug target mutation, drug target overexpression, modifications to drug uptake and efflux, activation of stress response pathways, and biofilm formation are only a few of the molecular drug-resistance mechanisms that *C. auris* has evolved (Figure 1) [9].

*Candida* spp. can become up to 1000 times more resistant to all currently known antifungals by clumping together and creating a biofilm [9].

The two components of the Beta(1,3)D-glucan synthase, the FKS1 and FKS2 genes, encode beta(1,3)D-glucan, a crucial part of the fungal cell wall [63]. This enzyme is inhibited by echinocandins, which lowers the amount of glucans in the cell wall [63]. The same two areas of FKS1 and FKS2 were the site of many echinocandin-resistant mutations in *C. albicans* and other non-auris species [53]. They were, therefore, designated as “hot-spots” 1 and 2 (HS1 and HS2) [53]. Molecular chaperones from the Hsp90 family are some of the major regulators of fungal biofilm dispersion, antibiotic tolerance, and cell wall remodeling [52]. These proteins have been shown to support stress responses associated with the administration of azoles and cell wall integrity signaling in *C. auris*, which has led to the evolution of drug resistance [52].

Efflux pumps, which are transport channels in the cell membrane that eject chemicals that can endanger survival, are the first weapon in this pathogenic yeast’s armory [53]. Notably, *C. auris* has two main efflux pumps—the Major Facilitator Superfamily transporters and the ATP Binding Cassette—that are connected to antifungal resistance [53]. These pumps were initially discovered because of their similarity to the efflux pump genes of *C. albicans*, and they are most frequently linked to azole resistance [53].

ERG11, an ergosterol gene essential to the formation of the cell membrane in *C. auris*, is a second gene of note concerning antifungal resistance [54]. The enzyme lanosterol 14-alpha-demethylase, which transforms lanosterol into ergosterol for cell membrane integrity and shape, is particularly encoded by ERG11 [54]. Increased azole resistance is linked to mutations in ERG11, with three main “hot spot” areas of the gene showing the highest frequency of mutations [54]. The strains of *C. auris* most commonly attributed to the enhanced resistance had mutations Y132F or K143R [54].

### 7.3. Future Perspective

The creation of effective medication combinations offers an alternative to clinical therapy because there are few therapeutic alternatives for *C. auris* [64]. By utilizing diverse modes of action, medicines in conjunction with azole have the potential to provide synergistic effects that enhance treatment efficacy and overcome drug resistance to azole in *C. auris* [64].

When micafungin monotherapy is ineffective in treating *C. auris* infection, combined treatment with amphotericin B may be useful as a second-line strategy [65].

The majority of antifungal medications are toxic, and fungal diseases are becoming resistant to less toxic medications, such fluconazole [66]. These issues point to the necessity of developing fresh treatment modalities for fungus infections [66]. One tactic that has been investigated throughout the years is combination therapy [66]. When antifungal drugs are combined with recently discovered antifungal compounds or phytochemicals, the results can include greater efficacy, a wider range of action, and shorter therapy duration [66].

The antibacterial properties of natural plant extracts, particularly essential oils, have drawn the attention of pharmaceutical industry experts [67]. Four substances—methyleugenol, carvacrol, thymol, and eugenol—have garnered much interest [67]. These substances have demonstrated strong in vitro antivirulence and fungicidal properties against various species of Candida [67]. Thymol and carvacrol have been found to inhibit Candida species’ hyphae and biofilm production [67]. It has been demonstrated that carvacrol is useful in lowering the expression of the Secreted Aspartyl Proteinase gene in isolates of *C. albicans* that are susceptible and resistant, with a greater impact on the resistant isolates [67].

In 28% of *C. auris* strains, the combination of carvacrol and widely used antifungal medications, such as amphotericin and nystatin, demonstrated a synergistic effect [66].

Orthologs of a number of *C. albicans* cell wall proteins, including those only expressed on *C. albicans* hyphae, have been reported to be expressed by *C. auris* [68]. HIL proteins are present in *C. auris*, and these proteins may make up a prime target for immunotherapeutic antibodies [68]. Out of the four clades of *C. auris*, at least three of the HIL proteins are anticipated to be surface exposed and have a high Tm score when compared to Cal-Hyr1p [68]. The fifth epitope on the HIL protein, which is highly immunogenic, surface accessible, and conserved, is found in all four clades of *C. auris* [68]. Since monoclonal antibodies often have a lengthy half-life in humans, the use of antibodies can be either therapeutic with antifungal medication or as a preventive intervention in high-risk patients [68].

## 8. Conclusions

It has been shown that *C. auris* can spread quickly both within and between healthcare facilities across different geographic locations. The global spread of infections presents a serious obstacle to public health and infection control initiatives. Since *C. auris* infections can be confused with other fungal infections, diagnosing them can be difficult. It might not always be adequately detected by conventional diagnostic techniques, which could cause delays in receiving the right care and possibly even epidemics. The resistance of *C. auris* to certain antifungal drugs that are frequently used to treat Candida infections is a matter of concern. Because of this, treatment becomes more challenging, which prolongs sickness, raises healthcare expenses, and raises mortality rates.

**Figure 2:**
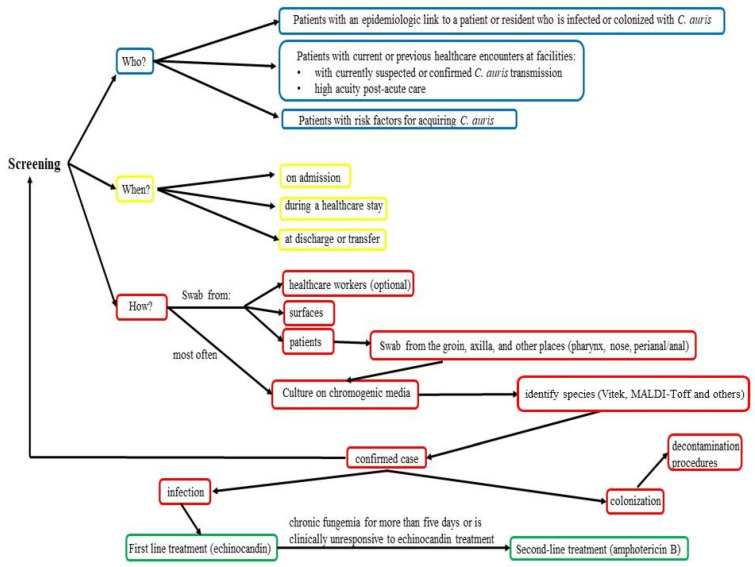
Prevention and treatment of *C. auris* infection [69].

High death rates have been linked to *C. auris* infections, especially in patients with weakened immune systems or underlying medical problems. Improving patient outcomes requires prompt identification and proper treatment (Figure 2).

It has been demonstrated that *C. auris* persists in the clinical setting, making infection control strategies difficult. This increases the danger of transmission in hospital settings and includes resistance to standard disinfectants and the capacity to persist on surfaces for extended periods.

To better understand and combat this multidrug-resistant fungal pathogen, research activities, infection control procedures, and surveillance are all made more crucial by the advent of *C. auris*.

## Figures and Tables

**Table 1:** Risk factors of *C. auris* infection [6,7,39].

Clinical Risk Factors	Non-Clinical Risk Factors	Medication-Related Risk Factors
Diabetes mellitus	Prolonged ICU stay	Corticosteroids
Renal failure	Age > 60	Presence of a central venous catheter
Chronic kidney disease	Obesity	Mechanical ventilation and/or intubation
Urinary tract infections	Male sex	Dialysis
Cardiovascular diseases	Surgery within 30 days	Colonized digital thermometers
Hematologic malignancy	Blood transfusion	Tocilizumab
Hypertension	Indwelling urinary catheter	Interleukin-6 receptor inhibitors
Ventilator-associated pneumonia		Invasive hemodynamic monitoring
HIV infection		Broad-spectrum antibiotics or previous exposure to antifungal agents within 30 days
Solid tumors		Incorrect use of PPE
Concomitant bacteremia or candiduria		Chemotherapy
		Parenteral nutrition

**Table 2:** Symptoms and signs of *C. auris* infection.

Possible Symptoms of *C. auris* Infection
Lethargy (extreme tiredness)
Pain, pressure, or feeling of fullness in the ear
Itching
Chills
**Possible Signs of *C. auris* Infection**
Low blood pressure
Tachycardia
Hypothermia
Fever

**Table 3:** Growth characteristics of C. auris and similar Candida spp.

Characteristics	*C. auris*	*C. haemulonii*	*C. duobushaemulonii*	*C. pseudohaemulonii*
Growth on Sabouraud with dextrose	+	−	−	−
Growth on Sabouraud with dulcitol	+	−	−	−
Growth on Sabouraud with manitol	+	−	−	−
Growth in 60% glucose	−	−	+	ND
Growth in vitamin-free medium	+	−	−	ND
Growth at 37 °C	+	−	+	+
Growth at 40 °C	+	−	−	−
Assimilation of L-Sorbose	−	−	+	+
Assimilation of Arbutin	ND	−	+	ND

Abbreviation: + positive, − negative, ND not determined. Adapted after [46,54,55].

**Table 4:** Aspect on different culture media of *C. auris* and other frequently identified Candida species (adapted after [57]).

Culture Medium	*C. auris*	*C. albicans*	*C. parapsilosis*	*C. glabrata*
Sabouraud dextrose agar **Medium**	White to cream			
BrillianceTM Candida Agar **Medium**	Beige to pink	Green	Beige/yellow/brown	Beige/yellow/brown
CHROMagarTM Candida **Medium**	Pale pink	Green	White, pale pink, or light lavender	Dark pink to purple

**Table 5:** *C. auris* misidentification through various laboratory methods. (Adapted after [1,14]).

Laboratory Identification Method	Organism Misidentification
All methods	*Candida haemulonii*
API 20C	*Rhodotorula glutinis* *Candida sake*
API ID 32C	*Candida intermedia* *Candida sake* *Saccharomyces kluyveri*
RapID Yeast Plus	*Candida parapsilosis*
Vitek2	*Candida duobushaemulonii* *Candida famata*
BD Phoenix yeast identification	*Candida catenulata*
MicroScan	*Candida famata* *Candida guilliermondii* *Candida lusitaniae* *Candida parapsilosis* *Candida catenulata*

**Table 6:** Proposed resistance breakpoints.

Antifungal	Proposed Resistance Breakpoint (µg/mL)
Fluconazole	≥32
Amphotericin B	≥2
Anidulafungin	≥4
Caspofungin	≥2
Micafungin	≥4

**Table 7:** Comparative doses of antifungal [60,61,62].

Echinocandin Drug	*C. auris*	Non-Auris *Candida* spp.
Anidulafungin	Loading dose 200 mg IV, then 100 mg IV daily	200 mg × 1 for first day, followed by 100 mg × 1
Caspofungin	Loading dose 70 mg IV, then 50 mg IV daily	70 mg × 1 for first day, followed by 50 mg × 1 (weight ≤ 80 kg) 70 mg × 1 (weight > 80 kg)
Micafungin	100 mg IV daily	Standard dose: 100 mg × 1 (weight > 40 kg) 2 mg/kg × 1 in patients weighing < 40 kg Increased exposure dose: 200 mg × 1 (weight > 40 kg) 4 mg/kg × 1 in patients weighing < 40 kg
